# Investigation of physical playfulness in physiotherapy students

**DOI:** 10.1186/s12909-023-04618-1

**Published:** 2023-09-08

**Authors:** Hayriye Kul Karaali, Ozlem Ozcan

**Affiliations:** Faculty of Health Sciences, Department of Physiotherapy and Rehabilitation, Manisa Celal Bayar University, Uncubozkoy Health Campus,. 5526 Street. No:8/4, Manisa, Turkey

**Keywords:** Play, Physical activity, Physiotherapy, Students, Children

## Abstract

**Background:**

Physiotherapists show a positive attitude towards playing games in order to be a role model for pediatric patients and to increase the success of therapy. The aim of the present study is to investigate the physical playfulness of physiotherapy students and the relationship of physical playfulness and individual and environmental factors.

**Methods:**

The sociodemographic data, regular physical activity habits of the students were examined as well as their computer game playing status and duration. “Attitudes of 18–22 Age Adults for Playing Games That Contain Physical Activity” scale was used for assessing playfulness.

**Results:**

A total of 268 students participated in the study. Among the game proneness scale subsections, the highest score was obtained in the Social Adjustment while the lowest scores were obtained in the “Desire to Play Game” and “To take pleasure from playing game” subsections. Male students scored higher in “Risk Taking and “To take pleasure from playing game” in comparison with the female students. There was a statistically significant difference between physical activity habits and “Game Compassion”, “Risk Taking”, “Social Adjustment” and “To take pleasure from playing game scores”.

**Conclusion:**

Physiotherapy students were found to be more playful, particularly in terms of social adaptation. Men take more risks in the plays and also play the games more enjoyable. Students with regular physical activity habits were more playful overall. The present study suggests that monitoring physical activity levels, computer games types might be beneficial for evaluating the playfulness.

## Background

From a biopsychosocial point of view, one of the features that support the social aspect of human is playing and being playful [1, 2]. Buytendijk (1933) defines the game as a certain type of playful movement with rules [3]. Plays are a variety of activities promoting the maturation and development of necessary skills in chilhood [4, 5]. Games enable the development of their communication and motor skills as well as they increase emotional skills and social interactions [1, 6]. Games has a crucial role in maximizing independence and expanding relevant individual goals in children. Accordingly, games also have a positive effect on the treatment process of children who are functionally dependent [4, 5].

Many types of games are available and one of them is physical activity games [7]. Such games present a higher intensity of physical activity than resting metabolic rate (running, jumping, etc.) [8]. Playfulness is defined as “an individual difference variable that allows the person to frame or reframe everyday situations in such a way that they experience them as entertaining, and/or intellectually stimulating, and/or personally interesting” [1]. Playfulness has a number of psychological and physiological benefits including problem solving, physical activity, and imagination [1, 9].

In recent years, it was stated that the participation in physical activity among university students has decreased with the intensive use of technology such as mobile phones and computer games [10–12]. Physical activity has an important place in the process of maintaining health and preventing diseases. In order to develop healthy behaviors, barrier and facilitator factors affecting physical activity participation are currently investigated. The fact that funny activity is considered among the factors that increase physical activity participation [13–15]. On the other hand, game increases the motivation of the individual when combined with physical activity because it is funny [16]. It has been emphasized in the current publications that use of exergaming (virtual exercise training game) increases the compliance of individuals to treatment of various diseases such as obesity, dementia and respiratory problems [17–19].

In physiotherapy training, some movements may be painful or create tension [20]. The fact that repetitive compulsory activities such as exercise is boring for children diagnosed with acute and chronic diseases and it may be difficult for these children to focus on treatment. Making these therapeutic activities enjoyable increases the success of the treatment [21, 22]. In a study, a group of patients with CP who received physiotherapy since childhood were asked about the factors that facilitated their participation in treatment. The patients stated that the fun of the treatment increased their motivation and participation in the treatment [20]. Furthermore, physiotherapists strengthen their communication with the child while transforming the activity to the game compatible with their purpose [2, 3, 21, 22,]. There is a need for integrating physical activity games with therapy for children receiving physiotherapy and rehabilitation services to be able to perform functional activities in an independent manner. Physiotherapists are one of the professional groups that observe children most frequently in their career [23–26]. Therefore, it is important for physiotherapists to show a positive attitude towards playing games both in order to be a role model for pediatric patients and to increase the success in the therapy [1].

An important factor in physical activity games is the intensity of the activity changing moderate to vigorous [27]. There are many individual and environmental factors that may affect intensity. One of them is that individuals need an ability to regulate their body temperature during the game. According to this hypothesis, children play games requiring less-intense physical activity in hot weathers [28]. In addition, gender has shown as a factor that will affect the severity of physical activity. The studies show that boys prefer games with more physical activity intensity because they spend more time with their fathers [8].

Therefore, the aim of our study is to investigate the playfulness of physiotherapy students and the relationship of playfulness and individual with environmental factors.

## Methods

Physiotherapy and Rehabilitation Department students with an age range of 18–22 years participated in the present descriptive study. Microsoft Forms program was used for creating voluntary consent form which contains information about the study, data registration form used in the evaluation and Attitudes of 18–22 Age Adults For Playing Games That Contain Physical Activity Scale. The students were informed about the study before participation. Students who volunteered to participate were asked to fill in the evaluation form that was provided via a link. It was assured by form design that all questions had to be answered and it was not possible to change a given answer and each student could log in to the form only once by using a username and password that is created uniquely for themselves.

### Inclusion criteria

Study participants were the first, second, third and fourth-year students of physiotherapy and rehabilitation department at Faculty of Health Sciences, Manisa Celal Bayar University. The voluntary students between the ages of 18–22 years were included in the study.

### Exclusion criteria

The forms of the students who were informed about the study and were voluntary participation, was reviewed. The forms of students under 18 and over 22 years are excluded (n = 12 students).

### Data Registration Form

The data form consisted of questions regarding age, gender, height, weight, geographic region of residence, regular physical activity habits, and computer game playing status of the participants. The daily time (hours) spent for playing computer games was asked to the students who play computer games. We questioned only whether the students had regular physical activity habits in accordance with physical activity recommendations for the 18–64 age group [29]. We calculated body mass index (BMI) that is calculated by person’s weight in kilograms divided by the square of height in meters [30].

### Game proneness scale: attitudes of 18–22 age adults for playing Games that Contain Physical Activity

The scale consists of 5 sub-dimensions and 25 questions as follows: game compassion (questions 9,10,13,14,15,16), risk taking (questions 3, 4, 8, 19, 20), social adjustment (questions 6, 7, 11, 17, 18, 22), desire to play game (questions 1, 2, 5, 12) and taking pleasure (questions 21, 23, 24, 25). Items in the scale are evaluated with a five-point Likert-type scale as 1 point indicating “I strongly disagree” and 5 points “I strongly agree”. The validity and reliability study was performed by Hazar 2015. The Cronbach’s alpha coefficients of the sub-dimensions are 0.83, 0.86, 0.79, 0.72 and 0.81 for game compassion, risk taking, social adaptation, desire to play game and taking pleasure from playing game, respectively [31].

### Sample size calculation

Epi InfoTM 7.2.4.0 software was used to calculate sample size. A 95% confidence interval was used for determining the number of participants needed. It was calculated that the minimum sample size should include 242 participants. Population size (for finite population correction factor or fpc) (N): 650. Hypothesized % frequency of outcome factor in the population (p): 50%+/-5 and confidence limits as % of 100 (absolute +/- %)(d): 5%. Sample size n= [DEFF*Np(1-p)]/ [(d2/Z21-α/2*(N-1) + p*(1-p)].

### Statistical analysis

SPSS software (version 25.0) was used for performing statistical analyses. Analytical methods (Kolmogorov-Smirnov/Shapiro¬Wilk tests) were used to examine the distribution normality of the variables. The descriptive analyses were presented as mean ± standard deviation and descriptive statistics of categorical data were calculated as frequency and percentage values. In continuous data, t-test was used to compare independent groups with normal distribution. One-way ANOVA analysis was used for comparison between more than two groups and also post-hoc test Bonferroni analysis was performed. Pearson’s correlation test was used to examine the relationship between variables (age, BMI and daily time spent playing computer games) and scores. The results with a p-value below 0.05 were considered statistically significant.

## Results

Of totally 652 department students, 366 (56.13%) were not included in the study because they did not answer the online form and 6 (0.92%) ticked the option “I do not volunteer to participate in the study”. Twelve of the 280 volunteer students were excluded because they did not meet the criteria for the age range of 18–22 years. As a result, 268 department students participated in the study and filled out the online form. Since no missing data was found in the filled forms, all forms were considered to be valid. The average time to fill out the form was 8 min and 29 s.

The Cronbach’s α value of our study was found 0.89. Cronbach’s α value of sub-dimension scale ranged 0.78 to 0.62 (Game Compassion = 0.78, Risk Taking = 0.71, Social Adjustment = 0.75, Desire To Play Game = 0.70, Taking Pleasure = 0.62). These values were acceptable.

76.5% (n = 205) of the students were female and 23.5% (n = 63) were male. The mean age was 20.8 ± 1.2 years and the mean body mass index was 22.1 ± 3.8 kg/m2. It was seen that most of the students (46.6%) were living in the Aegean Region followed by Marmara by 22.0%, Mediterranean by 9.0%, Central Anatolia by 8.2%, Black Sea by 6.0%, Eastern Anatolia by 4.5% and Southeast Anatolia by 3.7%. While 55.2% of the participant group stated that they did regular physical activity, 44.8% of them were not doing. Of the students, 34.7% (n = 93) stated that they played computer games averagely 2.4 ± 2.1 h (See Table 1).

**Table 1:** Descriptive Data of Students

Variables (N = 268)	Number (n)/Percent (%) Mean ± standart deviation (Min-Max)
**Age (years)**	20.8 ± 1.2 (18–22)
**Gender**	
Female Male	(n = 205) / (76.5) (n = 63) / (23.5)
**BMI (kg/m** ^**2**^ **)**	22.1 ± 3.8 (12.4–36.9)
**Region of residence**	
Aegean Marmara Mediterranean Central Anatolia Southeastern Anatolia Eastern Anatolia Black Sea	125 (46.6) 59 (22.0) 24 (9.0) 22 (8.2) 10 (3.7) 12 (4.5) 16 (6.0)
**Physical activity**	
Yes No	148 (55.2) 120 (44.8)
**Playing computer games**	
Yes No	93 (34.7) 175 (65.3)
**Daily time spent playing computer games (Hours/Day)**	2.4 ± 2.1 (1–15)

BMI:Body mass index

On the basis of mean score, it was found in the sub-dimensions of the game proneness scale that the participants gained the highest score in the sub-dimension of social adjustment (25.2 ± 3.1), followed by “risk taking” (15.0 ± 3.7), “game compassion” (14.8 ± 2.5), “desire to play game” (13.6 ± 3.7) and “to take pleasure from playing game” (13.6 ± 2.4), respectively (See Table 2). It was found that male scores in the dimensions of “risk tasking” (p = 0.005) and “to take pleasure from playing game” (p = 0.008) were significantly higher than those of females. The scores obtained from the dimensions of game compassion (p < 0.001), risk taking (p < 0.001), social adjustment (p = 0.001), to take pleasure from playing game (p < 0.001) by those with physical activity habits were significantly higher than those without physical activity habits.

**Table 2:** Game Proneness Scale: Score of Attitudes of 18–22 Age Adults For Playing Games That Contain Physical Activity Scale

Subtitles of Scale	Mean ± standart deviation
Game Compassion	14.8 ± 2.5
Risk Taking	15.0 ± 3.7
Social Adjustment	25.2 ± 3.1
Desire to Play Game	13.6 ± 3.7

The scores from the dimensions of game compassion (p = 0.008), risk taking (p = 0.005), to take pleasure from playing game (p = 0.001) were found to be significantly higher in the computer game players than those who were not players (See Table 3).

**Table 3:** Comparing Game Proneness Scale scores between demographical variables

	Game Compassion			Risk Taking			Social Adjustment			Desire to Play Game			To take pleasure from playing game		
Variables	*X ± SD*	p	t	*X ± SD*	p	t	*X ± SD*	p	t	*X ± SD*	p	t	*X ± SD*	p	t
Gender*															
Female (n = 205)	14.7 ± 2.5	0.116	1.575	14.6 ± 3.4	**0.005**	**3.284**	25.4 ± 2.9	0.238	-1.182	13.3 ± 3.5	0.055	1.929	13.4 ± 2.4	**0.008**	**2.673**
Male (n = 63)	15.2 ± 2.5			16.4 ± 4.4			24.8 ± 3.7			14.4 ± 4.2			14.3 ± 2.5		
Physical activity*															
No (n = 120)	14.1 ± 2.4	**p < 0.001**	**-3.893**	14.1 ± 3.2	**p < 0.001**	**3.799**	24.6 ± 3.1	**0.001**	**-3.332**	13.1 ± 3.4	0.077	-1.775	13.0 ± 2.3	**p < 0.001**	**-3.750**
Yes (n = 148)	15.3 ± 2.5			15.8 ± 4.0			25.8 ± 3.0			13.9 ± 3.9			14.1 ± 2.5		
Playing computer games*															
No (n = 175)	14.5 ± 2.5	**0.008**	**-2.680**	14.6 ± 3.5	**0.005**	**-2.838**	25.2 ± 3.0	0.726	-0.351	13.3 ± 3.3	0.064	-1.866	13.2 ± 2.4	**0.001**	-3.518
Yes (n = 93)	15.3 ± 2.3			15.9 ± 4.1			25.3 ± 3.3			14.2 ± 4.2			14.3 ± 1.3		
Region of residence**	***X ± SD***	**p**	**F**	***X ± SD***	**p**	**F**	***X ± SD***	**p**	**F**	***X ± SD***	**p**	**F**	***X ± SD***	**p**	**F**
Aegean (n = 125)	14.9 ± 2.6	0.427	0.427	15.1 ± 4.1	0.688	0.688	25.3 ± 3.0	0.652	0.652	13.5 ± 3.6	0.911	0.911	13.7 ± 2.5	0.958	0.958
Marmara (n = 59)	14.4 ± 2.6			14.6 ± 3.5			24.9 ± 3.7			13.6 ± 3.6			13.6 ± 2.3		
Central Anatolia (n = 22)	15.3 ± 2.4			15.6 ± 3.6			26.3 ± 1.7			13.8 ± 3.8			13.4 ± 2.9		
Southeastern Anatolia (n = 10)	13.9 ± 2.7			14.9 ± 3.1			25.2 ± 2.2			14.9 ± 2.4			14.2 ± 2.6		
Eastern Anatolia (n = 12)	14.3 ± 1.9			13.5 ± 2.4			24.8 ± 3.6			13.8 ± 3.5			13.3 ± 2.2		
Mediterranean (n = 24)	15.4 ± 2.3			15.4 ± 3.6			25.5 ± 2.5			13.3 ± 3.6			13.4 ± 2.4		
Black Sea (n = 16)	14.5 ± 1.8			15.6 ± 3.4			24.8 ± 3.5			12.9 ± 5.0			13.3 ± 1.9		

* Independent t test and** one way ANOVA test was used and p < 0.05 was considered significant. Mean ± standart deviation:X ± SD

No correlation was found between subscore variables of student age and game proneness (game compassion: r = 0.030 and p = 0.623; risk taking: r= -0.085 and p = 0.167; social adjustment: r= -0.115 and p = 0.061; desire to play game: r= -0.029 and p = 0.641; to take pleasure from playing game: r = 0.012 and p = 0.846) (See Table 4).

**Table 4:** Correlation between age, BMI and playing computer games and Game Proneness Scale

Variables	Game Compassion	Risk Taking	Social Adjustment	Desire to Play Game	To take pleasure from playing game	
Age	r = 0.030; p = 0.623	r=-0.085; p = 0.167	r=-0.115; p = 0.061	r=-0.029; p = 0.641	r = 0.012 ; p = 0.846	
BMI	r = 0.030; p = 0.625	r = 0.072; p = 0.242	r = 0.072; p = 0.239	r = 0.095; p = 0.120	r = 0.112; p = 0.067	
Daily time spent playing computer games	r = 0.018; p = 0.867	r=-0.159; p = 0.135	r=-0.037; p = 0.726	r = 0.122; p = 0.250	r = 0.161; p = 0.130	

BMI: Body mass index ; Pearson’s Correlation analysis was used and p < 0.05 was considered significant. r; Pearson’s Correlation Coefficient

There was no correlation between subscores of student BMI and game proneness (game compassion: r = 0.030 and p = 0.625; risk taking: r = 0.072 and p = 0.242; social adjustment r = 0.072: and p = 0.239; desire to play game: r = 0.095 and p = 0.120; to take pleasure from playing game: r = 0.112 and p = 0.067) (See Table 4).

In addition, there is no correlation between the subscores of duration of playing computer games and game proneness of the students either (game compassion: r = 0.018 and p = 0.867; risk taking: r= -0.159 and p = 0.135; social adjustment: r= -0.037 and p = 0.726; desire to play game: r = 0.122 and p = 0.250; to take pleasure from playing game: r = 0.161 and p = 0.130) (See Table 4).

## Discussion

The present study was carried out to evaluate the game proneness attitude of undergraduate students of the Department of Physiotherapy and Rehabilitation. The results showed that students had high scores in terms of game compassion, risk taking while playing, social adjustment in the game, desire to play, and taking pleasure of playing the game. Males took higher risks and enjoyed the game more than females. In all aspects, those were doing regular physical activity showed more game proneness attitude than those who were not doing. It was observed that the students who were playing computer games had more game compassion and risk taking behavior in the game and they also took more pleasure from playing compared to those who were not playing computer games.

### Playfulness

In the present study that we evaluated the students from the Physiotherapy and Rehabilitation Department, it was seen that our students adapted to the environment more, took more risks, showed more desire to play and took more pleasure while playing games compared to the study of Öztürk [32] conducted with the students of the Department of Coaching and Sports Management. In our study, it was observed that Physiotherapy Department students were better at social adjustment in terms of game proneness while their passion for playing games was lower; however, it was observed in Öztürk’s study [32] that the passion of the students for playing games was at a higher level in contrast with their their social adjustment skills. This study also reported that the students of the coaching department are better than those of the sports management department in terms of showing social adjustment and taking pleasure from playing [32]. The fact that the coaching students were better in terms of social adjustment of our students may be resulting from the fact that they have to communicate with the children in both areas of profession. In terms of the passion for playing, the desire of physiotherapists to play games with children that include physical activity may be in order to include the children more in the rehabilitation program. We argue that this is the reason why our results were similar, because the students of the coaching department also work to involve the children in sports related to game compassion. Although high game proneness scores were mentioned in the study of Balcı et al. [33] conducted to evaluate preschool teachers, sufficient data could not be reached to compare with our group because the study was published as an abstract.

Since the questionnaire used in our study did not present normal values, it is also debatable how playful the physiotherapy students should be. In addition, there are many factors that affect playfulness such as gender, number of siblings, gender of siblings and the climate of their residence region [8, 28]. In our study, we only investigated the effect of gender and region of residence, 77.6% of our group lived in a region with a temperate climate in Turkey. Because of this reason, we concluded that there was no difference regarding the region of residence.

### Gender

The present study showed that men scored higher in-game risk-taking behavior and taking pleasure from game. In Öztürk’s study conducted with Coaching and Sports Management students, no gender difference was found.^11^ On the other hand, in the study conducted by Kaya et al. with Physical Education students, it was stated that males were more willing to play games [34]. An important point is that higher scores were usually seen more often in the male gender in game proneness studies conducted between the ages of 10–14 years [35, 36]. Hazar et al. (2017) stated that men were more directed to sportive activities in patriarchal societies and were more involved in out-of-school activities [37]. Our results were similar, although the age groups are close to the play period. The results of the study supported that the male gender enjoys playing games in adulthood and takes risks while playing games. The studies on child development in the literature indicated that boys are more injured while playing. Among the reasons for this, it is shown that boys are different from girls in terms of muscle strength and coordination [8, 28]. The review of the studies in this area reveal that there are studies showing that boys or girls are more playful and the effect of gender is still discussed in the literature. However, regarding taking risks in the game as one of the sub-dimensions of playfulness, there are many studies stating that boys participate in games more actively and take more risks than girls [8]. Similar to the results in the literature, we found that young male adults took more risks at the end of our study.

### Physical activity

In addition, the present study demonstrated that students with a regular physical activity habit were better at all sub-dimensions of game proneness than those who have not. Although a previous study with pre-school teaching students [33] in the literature did not support our results, another study conducted with physical education students [34] obtained results supporting our results. As Akarçeşme et al. showed comparing team sports and sedentary athletes in the age group of 10–14 years, all game proneness sub-scores of those who do sports were better [36]. It has been stated that people who take part in regular physical activity have a more positive, social and enjoyable personality and try to take part in different fun activities. It is also stated that people with this personality structure are more playful as a view supported by the results of our study as well [37].

### Limitation

While the studies we examined were students who were known to do sports, we only asked students if they did regular physical activity according to WHO recommendations. One of our limitations was that we did not examine the characteristics such as physical activity intensity and duration. In addition, the majority of our study subjects were female.

## Conclusion

In conclusion, the present study shows that our physiotherapist candidate students have a good level of game proneness. It is also noteworthy that male students and those who do regular physical activity have more positive attitudes towards playing games.

Thus, we conclude that it is important to develop approaches to ensure that female physiotherapist candidates are monitored in terms of playing attitude and physical activity participation throughout their undergraduate education in order to obtain more effective results from the physiotherapy and rehabilitation programs which they will apply to the pediatric patient, because they will frequently observe pediatric patients in their professional lives.

In the future, a more homogeneous group should be studied to investigate the effect of gender. In terms of climate change, there is a need for studies that will involve patients groups from various climates.

Our study was carried out with physiotherapy and rehabilitation students. Our students were the students who had an idea about the role of play and the importance of physical activity in communication with the child. Beside our students, we conclude that it is necessary to investigate how playful physiotherapists are while working with patients in the future.

## Data Availability

The datasets used and analysed during this study are included in this article and are available from the corresponding author on reasonable request.
